# IgA Vasculitis Following COVID-19 Vaccination

**DOI:** 10.7759/cureus.33938

**Published:** 2023-01-18

**Authors:** Elvana Rista, Arjana Strakosha, Kristi Saliaj, Florida Ymeri, Majlinda Ikonomi

**Affiliations:** 1 Department of Nephrology, Hygeia Hospital Tirana, Tirana, ALB; 2 Department of Nephrology, University Hospital Center Mother Theresa, Tirana, ALB; 3 Department of Histopathology, University Hospital Center Mother Theresa, Tirana, ALB

**Keywords:** autoimmunity, adult, vaccine, iga vasculitis, covid-19

## Abstract

As new variants of SARS-CoV-2 continue to emerge worldwide, countries are striving to fully vaccinate their population in a bid to prevent severe disease, subsequent hospitalizations, and the associated strain on their healthcare systems and death. In this context, there is growing evidence of rare, potential side effects associated with COVID-19 vaccines. IgA vasculitis is a systemic, IgA-mediated vasculitis characterized by palpable purpura, arthralgia, abdominal pain, and renal involvement. It is the most common type of vasculitis in childhood, sporadically affecting the adult population. However, there have been multiple reports of IgA vasculitis following vaccination against COVID-19. Herein, we present the case of a 72-year-old patient with palpable purpura that developed two weeks after receiving the Pfizer BioNTech vaccine. Laboratory investigations revealed elevated serum creatinine (2.6 mg/dL), macroalbuminuria (8.6 g/24 h), and macroscopic hematuria. Histopathological examination confirmed necrotizing vasculitis, and a diagnosis of IgA vasculitis was established. Considering the clinical presentation, the laboratory and histopathological findings, and the time interval between the vaccination and the development of symptoms, we strongly believe that IgA vasculitis in this patient arose as a side effect of the Pfizer BioNTech vaccine.

## Introduction

IgA vasculitis (Henoch-Schönlein purpura) is a systemic, small vessel vasculitis defined by IgA1-immune complex deposits primarily affecting the skin, musculoskeletal, gastrointestinal, and renal systems [1-5]. The estimated annual incidence of IgA vasculitis is 3-26.7/100,000 in the pediatric population and 0.8-1.8/100,000 in adults, underscoring the low prevalence of the disorder among the adult population [3,6]. Viral and bacterial infections are considered to be essential triggers, in addition to a combination of genetic susceptibility and environmental factors [1,3,5,7-9]. Common clinical manifestations include palpable purpura, arthralgia, abdominal pain, gastrointestinal symptoms, and renal dysfunction [1,3,5-9]. Treatment approaches for mild cases are mainly supportive, while management of severe cases with organ involvement includes a corticosteroid and immunosuppressive therapies [3,5-9].

As vaccination campaigns against COVID-19 have steadily expanded globally, with vaccination numbers consistently rising and implementing "booster" programs, several rare complications, possibly associated with COVID-19 vaccines, have been observed. The most common side effects of COVID-19 vaccines typically include pain, redness, soreness at the injection site, fever, fatigue, and headaches. Nevertheless, infrequent complications have been reported, including anaphylaxis, myocarditis, pericarditis, rhabdomyolysis, Guillain-Barré syndrome, Bell's Palsy, and transverse myelitis [10]. IgA vasculitis has emerged as a potential rare complication of vaccines against COVID-19, with multiple reports noting its occurrence following the administration of different vaccines. Herein, we present the case of a 72-year-old male patient that developed IgA vasculitis two weeks after receiving the Pfizer BioNTech vaccine.

## Case presentation

A 72-year-old male patient was admitted to the Department of Nephrology on account of a five-day history of nephrotic range proteinuria, macroscopic hematuria, generalized arthralgia, and palpable purpuric lesions in the inferior extremities. He reported that these complaints first arose with the development of palpable purpura in his lower limbs, which prompted a consult with a dermatologist. His past medical history was significant for arterial hypertension, chronic atrial fibrillation under treatment with rivaroxaban, heart failure, and type II diabetes mellitus. The patient disclosed having received the second dose of the Pfizer-BioNTech vaccine against COVID-19 two weeks prior to the appearance of the cutaneous lesions. The patient was initially started on topical therapy with 1% hydrocortisone and emollient creams. Renal function tests (RFTs) were ordered, revealing elevated levels of serum creatinine 1.2 mg/dL (normal range 0.7-1.1 mg/dL) and microscopic hematuria (10-15 RBC/HPF), evident in his urinary sediment. In view of his compromised renal function, additional laboratory examinations, including comprehensive metabolic and immunological panels, were scheduled, and the patient was referred to the Department of Nephrology.

**Figure 1:**
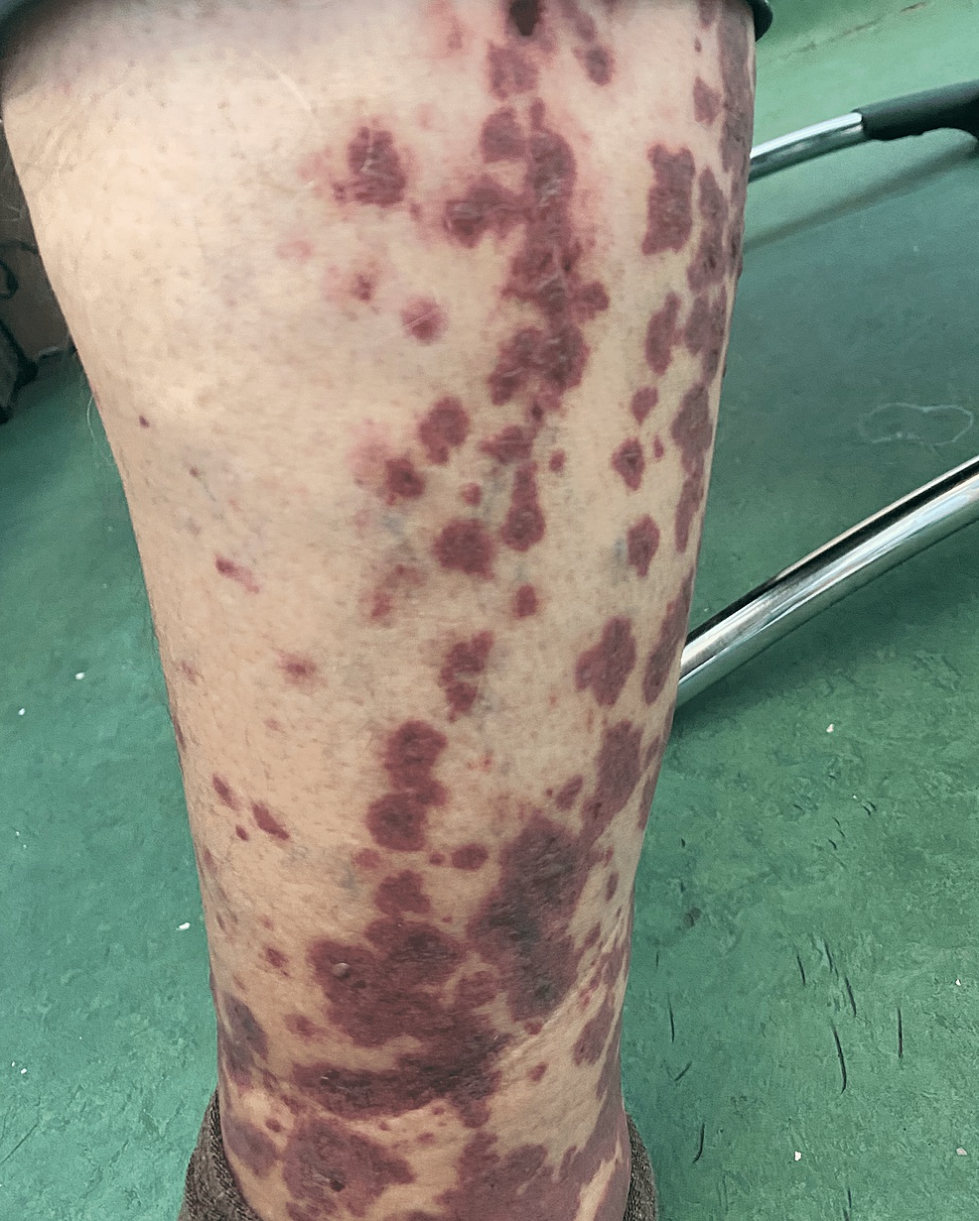
Palpable purpura. Dark red and purple, elevated, firm, confluent round papules in the lower limbs with signs of erosion and necrosis.

**Figure 2:**
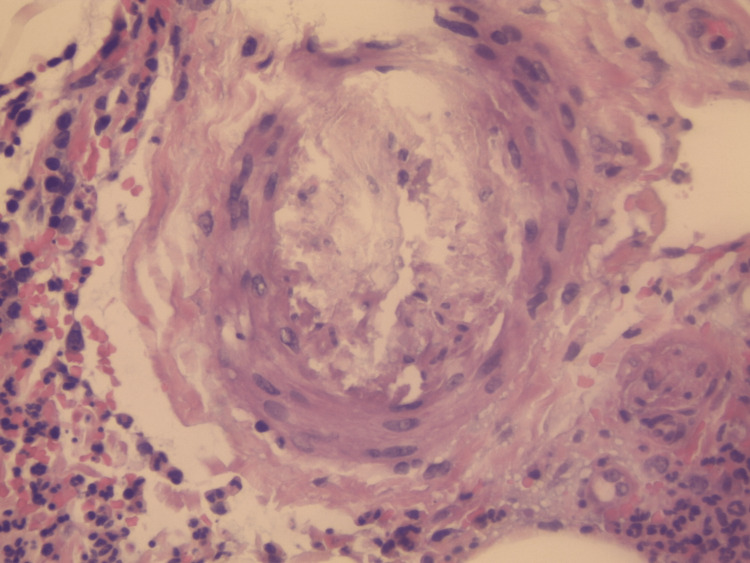
Leukocytoclastic vasculitis (H&E staining) The vessel walls are thickened due to adipose tissue and fibrin deposits, as well as the presence of diffuse lymphoplasmacytic and neutrophilic infiltrates. Presence of endothelial edema, extravasated RBCs, and polymorphonuclear inflammatory cells involving the superficial dermis layer.

**Figure 3:**
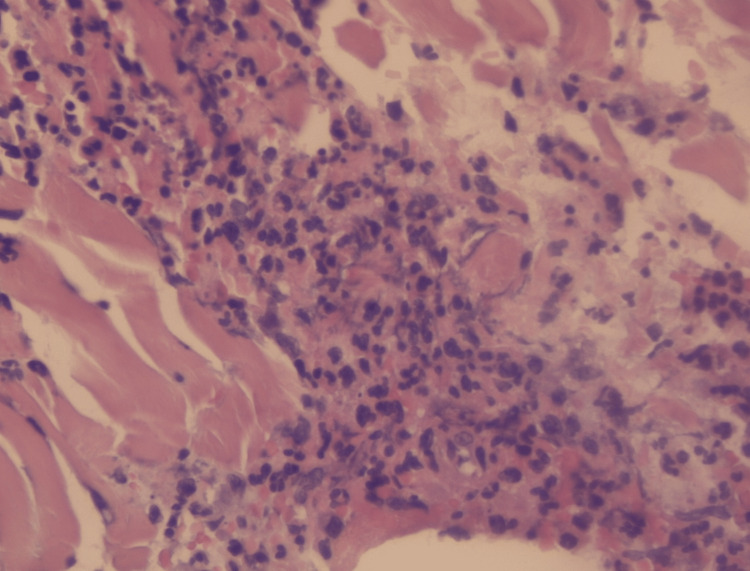
Leukocytoclastic vasculitis (H&E staining) The vessel walls are thickened due to adipose tissue and fibrin deposits, as well as the presence of diffuse lymphoplasmacytic and neutrophilic infiltrates. Presence of endothelial edema, extravasated RBCs, and polymorphonuclear inflammatory cells involving the superficial dermis layer.

**Figure 4:**
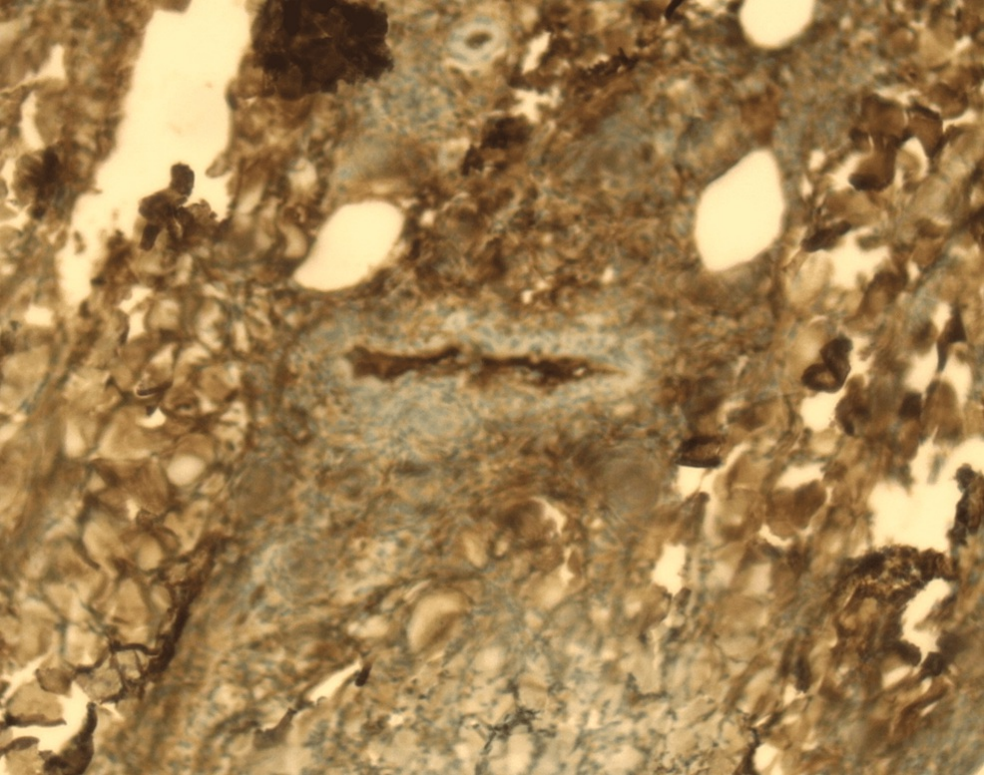
Leukocytoclastic vasculitis (immunohistochemistry). Positive immunohistochemistry testing for IgA.

**Figure 5:**
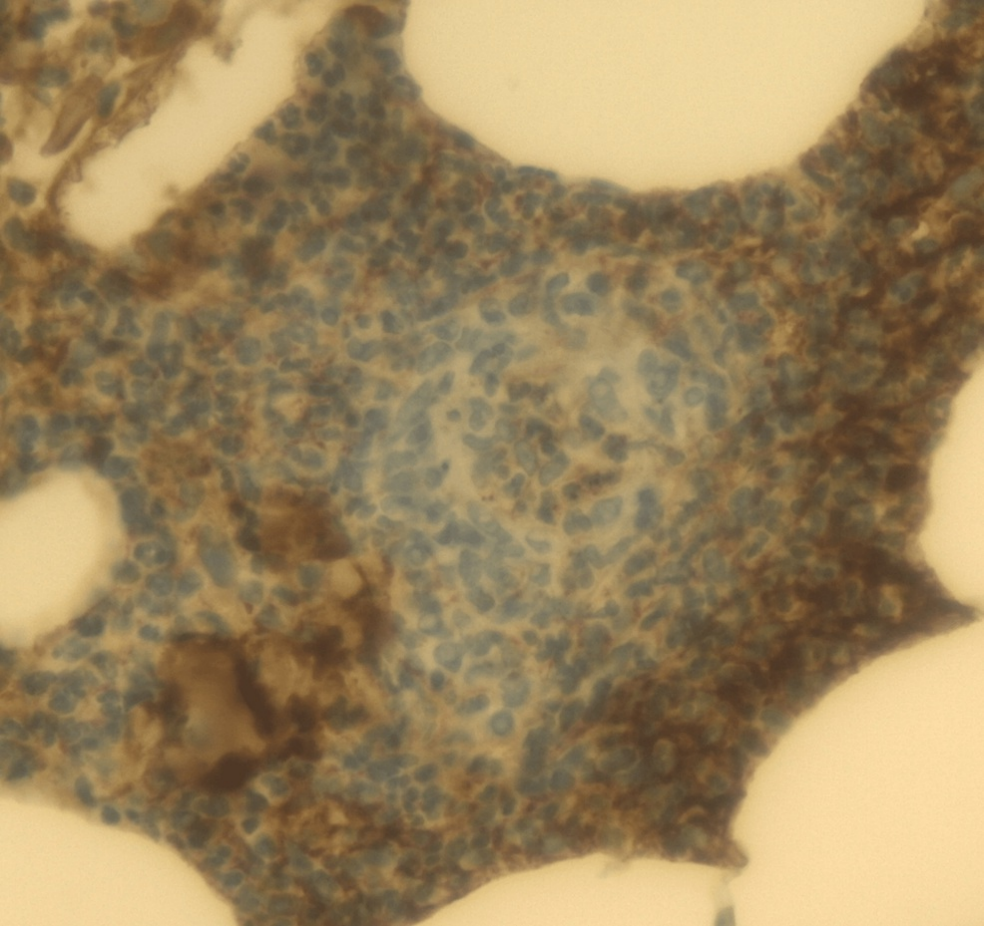
Leukocytoclastic vasculitis (immunohistochemistry). Positive immunohistochemistry testing for IgA.

On physical examination, palpable purpura and slight pretibial edema were noted in the inferior extremities (Figure 1). The rest of his physical examination was unremarkable. During the course of his stay, the purpuric lesions persisted, and 24-hour urine collection showed nephrotic range proteinuria (8.6 g/24 h) and macroscopic hematuria with the presence of over 80% dysmorphic RBCs and serum creatinine levels rose to 2.6 mg/dL. Immunological assays identified elevated levels of serum IgA, decreased levels of C4, and negative p-ANCA and c-ANCA. The rest of the laboratory and imaging studies were within normal limits, including a pulmonary CT scan to rule out potential pulmonary involvement. Skin biopsy revealed necrotizing vasculitis (Figures 2-5).

**Figure 6:**
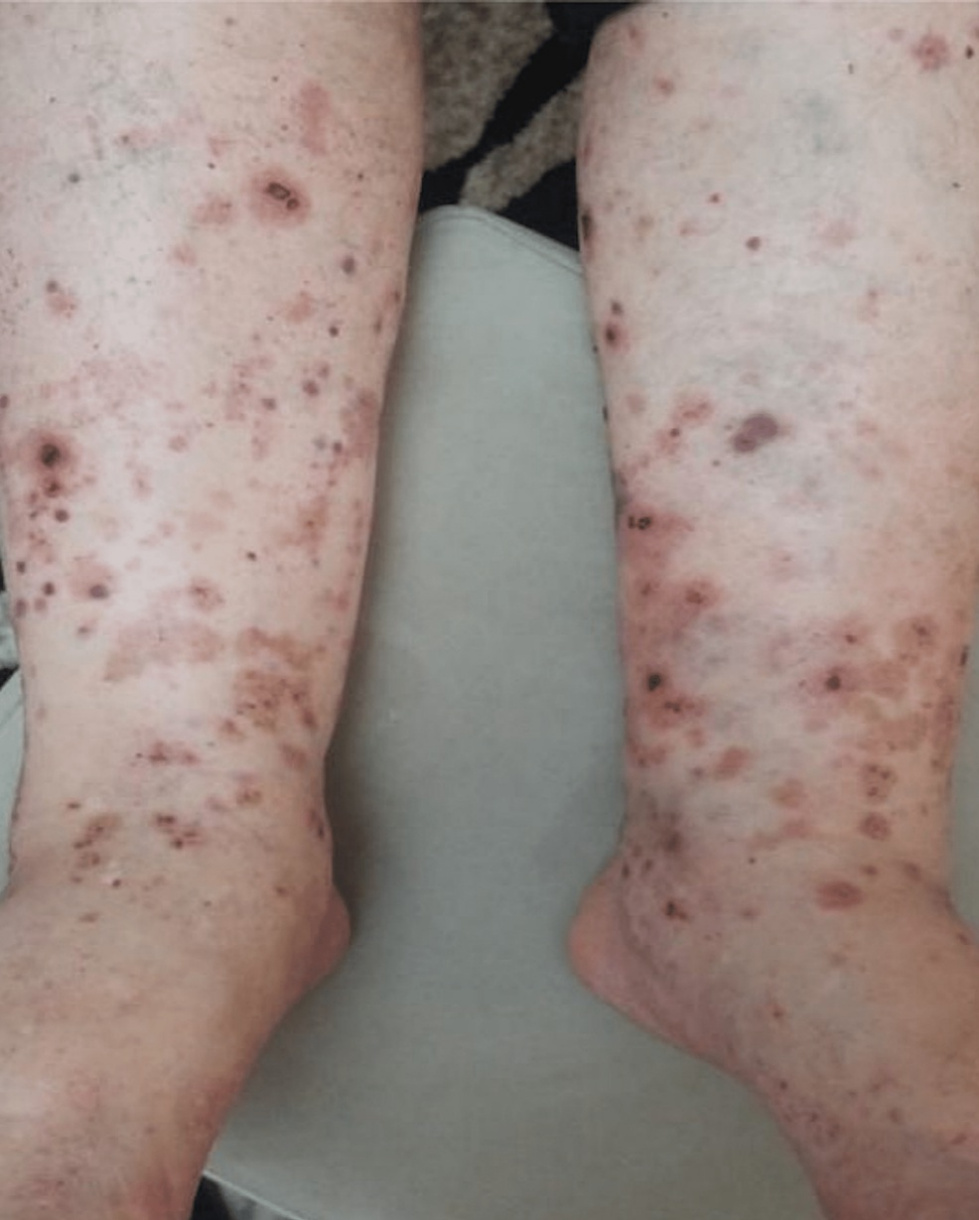
The purpuric rash following corticosteroid treatment.

In the context of the macroscopic hematuria, nephrotic range proteinuria and renal dysfunction, associated with palpable purpura in the inferior extremities, generalized arthralgia, abdominal pain, along with elevated levels of IgA serum protein electrophoresis and evidence of necrotizing vasculitis in the skin biopsy, pointed to IgA vasculitis (Henoch-Schönlein purpura). The patient initially received a three-day course of high-dose pulse methylprednisolone therapy, followed by an oral prednisone therapy of 60 mg daily. Dosing was optimized based on his comorbidities and treatment response. Cutaneous lesions along with renal function started improving rapidly within two weeks following the initiation of the corticosteroid therapy, with levels of serum creatinine and albuminuria decreasing to 2 mg/dL and 1.8 g/24h, respectively (Figure 6). 

We believe the disorder developed following the administration of the Pfizer BioNTech COVID-19 vaccine, a rare complication that has been previously reported in a few recent studies. 

## Discussion

An association between IgA vasculitis and vaccines against viral antigens is well-established, with reported cases of de novo IgA vasculitis, following vaccination against influenza, rabies, hepatitis A and B viruses, MMR vaccine, and pneumococcal and meningococcal disease [8-12]. Over the course of the past year, as vaccination programs were first introduced and vaccine rollout began worldwide, several cases of IgA vasculitis following the vaccination with Pfizer BioNTech, Moderna, AstraZeneca, and viral vector vaccines have been reported [10-18]. The pathological mechanisms underpinning this rare complication are yet to be fully elucidated. Nevertheless, several theories have emerged, postulating that a strong IgA1-mediated immune response against the spike protein or vaccine components, including the mRNA and the lipid packaging membrane, may be the culprits [15,16]. It has also been proposed that the additional release of cytokines further activates IgA1-producing B cells, leading to cutaneous and renal immune complex depositions and the development of IgA vasculitis [15]. Two weeks following his vaccination with the Pfizer BioNTech vaccine, our patient presented with bilateral palpable purpura in his lower limbs, and additional laboratory investigations denoted a renal involvement. This presentation was consistent with other reports in the literature that showed the time of presentation varied between 1 and 19 days [10-18], as well as previous studies underscoring the development of IgA vasculitis within 14 to 21 days following influenza immunization [10]. Given that symptoms associated with IgA vasculitis stem shortly after the administration of COVID-19 vaccines, other than a temporal association, current literature points to a potential causal relationship that remains to be proven. 

Treatment strategies for IgA vasculitis are optimized based on the clinical presentation and the extent of the systemic involvement. Mild cases are generally self-limiting, requiring only supportive measures, including acetaminophen and non-steroidal anti-inflammatory drugs (NSAIDs) that can be used to relieve fever and arthralgia [3,5-9]. Therapeutic options for moderate to severe cases characterized by renal and gastrointestinal involvement consist of corticosteroid therapy, immunomodulatory and immunosuppressive agents like mycophenolate mofetil, dapsone, azathioprine and rituximab, IV immunoglobulin therapy, plasmapheresis and medications like colchicine and ACE inhibitors in the setting of renal disease [3,5-9]. Based on the recently published data and our personal experience, the standard of care for most patients that develop IgA vasculitis with renal involvement following COVID-19 vaccination is corticosteroid therapy, providing excellent outcomes [10-18]. In severe cases, immunosuppressive therapy can also be used successfully [19,20]. Finally, we believe this case report to be of significant clinical relevance because IgA vasculitis predominantly affects the pediatric population, with adult patients representing only a minority of cases, generally associated with higher severity and poorer outcomes. However, our review of the current literature revealed that IgA vasculitis following COVID-19 vaccination was more prevalent in adults, as opposed to children, and responded well to treatment, with favorable outcomes.

## Conclusions

As evidence of infrequent complications following COVID-19 vaccination continues to mount, this case report adds to the accumulating literature on IgA vasculitis as a potential rare complication of COVID-19 vaccines. It is important to consider the development of de novo or recurrences of different autoimmune disorders, including IgA vasculitis, in patients presenting within a short time frame from administering a vaccine against COVID-19. Awareness of a possible link between IgA vasculitis and COVID-19 vaccines is essential for a timely diagnosis and prompt treatment. Supportive care and early initiation of corticosteroid or immunosuppressive therapy in severe cases are largely associated with excellent outcomes.
